# Transactions of the Provincial Med. and Surgical Association

**Published:** 1837-01

**Authors:** 


### ART. IX.

*Transactions of the Provincial Medical and Surgical Association.* Vol. IV.—*London and Worcester*, 1836. 8vo. pp. lxxvi. 578; plates.

THE present volume appears in every way to justify the opinions which we have already expressed of the importance and usefulness which the excellent Society from which it emanates seems destined to attain. Many of the papers are calculated to add to the sum of our knowledge, and to advance materially the progress of medical science; and we are especially gratified to observe that the attention paid to the subjects of Medical Topography and Medical Statistics is such as to lead to the anticipation of the best results. In the volume before us there are several papers belonging to these departments of medical enquiry, including the continuation of Dr. Forbes's Essay on the Medical Topography of the Land's-end; a paper on the Medical Statistics of Malvern and its immediate Vicinity, by Mr. Addison; Statistical Observations on the Medical Charities of England and Ireland, by Dr. Walker; with Hospital Reports by Messrs. Parsons, Ryland, Middlemore, and Johnson. To these, however, it is not our purpose to allude on the present occasion, further than to direct the attention of our readers to them, as not only containing details of extreme value, but presenting models that may be carefully kept in view by other observers in a department which is yet in its infancy among us. The remainder of the volume is occupied partly with the proceedings of the Anniversary Meeting held at Oxford in the year 1835, including the Address read upon that occasion by Dr. Prichard; and a Report upon the Fevers and other Diseases which prevailed at Amsterdam during the year 1834, by Dr. Nieuwenhuys, one of the foreign correspondents of the Association; and partly with a series of Essays and Cases on miscellaneous medical subjects.

Perhaps no duty more important devolves upon the council of the Association than the selection of the individual who is to deliver the annual Retrospective Addresses; for, if any value attaches to these productions, it must arise from their presenting a correct and faithful account of the progress made not only in one branch, but in the whole aggregate of science which in any way bears upon the advancement of medical knowledge. To accomplish this object effectually requires a combination of natural talent, acquired knowledge, and sound judgment, which perhaps falls to the lot of few individuals. Hitherto the Society has been fortunate in this respect, and we are glad to perceive that Dr. Prichard not only entertains opinions similar to those here expressed, but that he has also fulfilled the task allotted to him in strict conformity to them. If there is any fault to be found with his Address, it is the very uncommon one, that it betrays perhaps a deeper acquaintance with the medical literature of other countries than with that of our own.

No one can doubt that an extraordinary degree of intellectual activity prevails in the present age, and that there is a steady and rapid progress in medicine as well as in the other sciences, requiring some such periodical summary as these addresses afford. "If," says Dr. Prichard, "we enquire by what steps a transition has been made into the way of thinking and reasoning on medical subjects which characterizes the present time, we shall find that one main part of this revolution has consisted in a partial abandonment of theoretical systems. It required a long training of the human mind before it could be brought, in every department of science, to recognize the real boundaries of attainable knowledge. In no department are these boundaries more restricted than in medicine. Ultimate facts present themselves on every side: we neither know by what operation the most powerful morbific causes, such as miasmata, produce their effect upon the human body; nor how the most simple remedies act in restoring a healthy state. Every dose of jalap is given empirically, and is expected to purge merely on the ground of analogy, and because other doses of jalap have been already known to purge. How, then, in relation to subjects of this nature, can a doctrine of causes and effects, or (in other words) a system of theoretical pathology, be erected on a secure foundation?" It is, in fact, the abandonment, at least in part, of the speculative mode of studying our science, which so long held the most philosophical minds in thraldom, and the following out of other and more legitimate methods of enquiry, that characterizes the present era. It is now that we are for the first time bringing to bear upon medical science those grand principles of the inductive philosophy which can alone form the basis of any solid superstructure in this as well as in other departments of knowledge. The brilliant theories of former times, considered as general systems of pathology, are at the present time little better than mere matter of historical detail; interesting, indeed, as tracing the progress of the human mind, which, in its search after truth, is ever liable to be led away by every speculation which crosses its path, but still of no real avail in the attainment of the professed object of its pursuit. The futility of such speculations being at length felt and acknowledged, the cultivators of medicine then had recourse to the observing and accumulating of facts. These, however, of themselves, and as isolated truths, are comparatively of little importance; and, even when taken collectively, from their number and apparent discrepancy, lead only to confusion; a confusion which the records of each day tend but to increase. But we are now in a condition, by a cautious application of the principles of induction, to unravel many of their intricacies; and we hope that the Addresses at each succeeding anniversary of this Association will record the establishment of some of those real principles which are alone to constitute the data from which more general laws can be deduced, and the groundwork upon which the true system of pathology is hereafter to be constructed.

The first part of the Address before us contains a condensed view of the state of medical science previous to the present period; and though, after the excellent summaries which have been already given by his predecessors, the author might perhaps have passed over, or at least materially shortened, this the introductory part of his Address, without detriment to the objects of the Society, still we must admit that, viewed as a separate production, and not as forming part of a series, it possesses the appearance of a more finished and complete essay. With regard to the actual progress made during the past year, Dr. Prichard enters upon its consideration under four sections, viz.—1. History of Diseases, or Nosography; 2. Technical Investigation; 3. Necroscopy, and Pathological Anatomy; and, 4. Observations on the effects produced by particular agents. Various questions which have lately received elucidation are then entered into; but, for information upon these points, we must refer to the work itself.

The *report* of Dr. Nieuwenhuys, of Amsterdam, presents an interesting account of the state of disease in that city and its neighbourhood, during the year 1834, and more especially of the intermittent and remittent fevers, and of the epidemic scarlet fever, which then prevailed. The report is accompanied by some meteorological observations and statistical details, to which we may hereafter have occasion to refer. The account of the scarlatina, as it occurs in this marshy country, is worthy of consideration and comparison with the phenomena presented by the same disease in localities less exposed to vegetable miasms. Dr. Nieuwenhuys believes the origin and extension of the scarlet fever which prevailed at this period to have been epidemic rather than contagious, although he admits that it may become contagious under favorable circumstances. The epidemic nature of the disease upon this occasion is perhaps confirmed by the results of a table given by the author, which shews a rise, increase, and diminution of the scarlatina, nearly corresponding with the progressive changes of the intermittent and remittent fevers of the same period, and affords also some support to the opinions of those writers who consider the scarlet fever in its different forms to be merely a modification of ordinary fever, whether remittent, simple continued, or typhoid. Dr. Nieuwenhuys found the belladonna to be of little or no use as a preventive of the disease; the fever taking place in some instances where it had been employed, whilst it did not occur in others in which it was not had recourse to.

The essays and cases contained in the fourth part or section of the volume, are the following:—1st. *On the Physiology and Pathology of Intermittence*, by Dr. Cowan, of Bath; 2d. *A Memoir on the Closing of Moist Anatomical Preparations*, by Mr. Crosse, of Norwich; 3d. *bservations on the Effects of the Secale Cornutum*, by Mr. Chavasse, of Birmingham; 4th. *On the Unity of Organic Structure*, by Dr. Paris Dick, of Bristol; 5th. *On Hydrometra*, by Mr. Coley, of Bridgenorth; 6th. *Records of Ovarian Tumours*, by Dr. Barlow, of Bath. The cases are, one of *Neuralgia of the Uterus*, by Dr. Holbrook, of Crickhowell; a case of *Disease of the Pudendum*, by Mr. Brayne, of Banbury; a successful case of *Cæsarean Operation*, by Mr. Knowles, of Birmingham; and a case of *Fungoid Tumour and Melanosis*, by Dr. Norris, of Stourbridge.

Dr. Cowan's Observations on *Intermittence* deserve a careful and attentive consideration, constituting, as they do, what we believe to be the first attempt at the investigating of this subject in a correct and philosophical manner. The pathology of intermittence has hitherto been chiefly studied in relation to the fevers of marshy districts, and to some varieties of neuralgia; but in each of these cases in a very imperfect and unsatisfactory manner. This is perhaps, in part, to be attributed to the undue importance hitherto attached to the derangements, whether functional or otherwise, of the vascular system, and to the doctrines of inflammation, as forming the sole ground-work of our modern views of pathology. The attempt to refer every disease to an inflammatory process going on in some part or other of the animal system, and every morbid change which dissection reveals to the effects of this process upon the organization, has, we are convinced, tended much to retard the progress of our science. The phenomena of intermittence have, however, always constituted a stumbling-block in the way of this exclusive pathology; and even Broussais himself, with all his ingenuity, and all the force of his sophistry, has been unable to obviate the objections to the *Doctrine de la Médecine Physiologique* which the phenomena of intermittent fever present. Dr. Cowan, in the first place, examines the subject of intermittence physiologically; that is, in the natural and healthy condition of the animal system; having previously endeavoured to remove a source of confusion and error in which the subject has occasionally been involved, from a want of due discrimination between this property and that of periodicity. This latter he defines to be "the recurrence of certain phenomena, at either regular or irregular intervals, which are constantly reproduced by the renewed presence of the cause on which they originally depended; phenomena which would necessarily cease, were the same cause, or one capable of producing similar effects, never to return." Day and night, and summer and winter, are then referred to as affording instances of periodicity depending upon the regular action of an external cause. Intermittence is thus characterized: "By *intermittence* we understand a succession of similar or nearly similar phenomena, regular or irregular in their periods of activity or repose, not resulting from the repetition of influences from without, but from some functional peculiarity inherent in the organs themselves. By this definition we at once set aside the consideration of those annually recurring changes in the inorganic and vegetable world, arising from causes to which we have already referred; as well as all those healthy or morbid modifications of the functions of animal life which are the simple result of altered external conditions, on whose presence they essentially depend for both existence and reproduction." Excepting the functions of circulation, respiration, digestion, and generation, considered in themselves, from the operation of this principle, Dr. Cowan proceeds to shew that it is closely connected with the functions of the nervous system. But here we must allow him to speak for himself.

"One of the most remarkable peculiarities is the necessity for periods of *repose*, of cessation from movement, and of insensibility to external impressions. These periods, almost, if not entirely, absent in the first rudiments of animal life, become gradually prolonged and distinct as we ascend, and are additionally necessary in proportion as organization approaches its highest point of development. They are not, as in the examples we have hitherto examined, the result of certain external physical changes, (though, in the great majority of cases, they coincide with them), which, by their more or less regular recurrence, induce a corresponding increase or decrease of functional activity, but arise from a condition of organization itself; a condition only common to one portion of our frame, considered physiologically; viz. to the nervous system which presides over the functions of animal life. No one supposes that the circulation or respiration ever ceases to be active, although they are undoubtedly modified by a state of repose of the rest of the body. Should one or the other cease, life becomes impossible, and we can form no idea of a living being independently of their influence. But, when the moral and intellectual faculties, the power of locomotion, the voice, the sight, the hearing, the smell, and the touch, become inert, we never imagine that life is extinct, but regard this temporary cessation of all our voluntary functions as a natural consequence of their dependence on the activity of certain central portions of the nervous system, to which we tacitly concede the fact of intermittence, though often forgetting to view it in the light of a functional peculiarity." (P. 271.)

Having thus shewn, as we think satisfactorily, although, from the condensed manner in which Dr. Cowan expresses himself, somewhat obscurely, that the principle of intermittence is, considered in its manifestations in the state of health, an attribute of the nervous system, he proceeds to draw the inference that the phenomena of intermittence evidenced in the animal body when labouring under disease, are to be considered as connected with and resulting from some morbid change, some functional derangement of the same system. Now, although we are not quite prepared to admit that this inference is logically to be derived from the premises laid down, we are still inclined to think that the conclusion is in itself correct. It is certain that, in those cases in which intermittence is most marked, however they may differ amongst themselves, a mode of practice which is successful in one is more or less applicable to all, and commonly avails where other methods have previously failed. The phenomena of intermittence in the state of health so far differ from those observed in the state of disease, that, while the latter are marked by the continued exercise of the cerebral and nervous functions and the suspension of superadded phenomena, the former are characterized rather by the absence or imperfect fulfilment of these functions as they are found to occur in their active and healthy state. In either case, however, there is an evident tendency in the intermitting phenomena in a state of disease to become continued. Thus, in the case of the temporary suspension of the mental operations which occurs in health, as, for instance, during sleep, a more excited state of the functions of the brain and nervous system leads to the continuance of these organs in a state of activity, and a consequent suspension of the regular, and in this case natural, intermission. Again, in the phenomena of intermittence as they occur in morbid states,—in intermittent fever, for example,—we have the undue excitement, or otherwise altered condition, which prevails during the paroxysm, passing through all the stages of imperfect intermission, as shewn in remittent fevers of various degrees and types, until the intermission or suspension of superadded phenomena is completely lost in those febrile states which are commonly termed continued fever. Dr. Cowan has scarcely carried his observations to the extent which the state of our knowledge admits and the importance of these views demands. At the same time we are fully aware that the subject is involved in difficulties of no ordinary kind, requiring for their elucidation much patient research, together with considerable discretion in the admission or rejection of such facts as seem to bear upon it. We hope, however, notwithstanding these difficulties, that the next volume of the Transactions will shew that Dr. Cowan is disposed to undertake this investigation, and to prosecute the enquiry until its relation to several interesting points of pathology with which it is intimately connected shall have been fully ascertained.

Mr. Crosse's Memoir *on the Method of securely closing Moist Anatomical Preparations preserved in Spirit*, we must pass over, as, although well deserving the attention of the practical anatomist, it is not well calculated for analysis in our pages. It is a very valuable paper.

The observations on the effects of the *Secale Cornutum* by Mr. Chavasse are highly deserving of the attentive consideration of those who are engaged in the practice of midwifery. According to the experience of this gentleman, two momentous evils are to be feared from the incautious use of this powerful medicine, especially in first labours, to which Mr. Chavasse wishes it to be understood that his remarks more particularly apply. These are, first, the death of the child; and secondly, retention of the placenta, with hour-glass contraction of the uterus. Two cases are related, of which we select the following for quotation, as being the shorter.

"Mrs. H., a delicate woman, thirty-six years of age, had been some hours in labour, the presentation being natural, when the liquor amnii escaped, and the pains, which before were regular, and, though slow, increasing in frequency, became suspended for three-quarters of an hour; the child being lively and troublesome. The circumference of the os uteri was now equal to that of a crown-piece, and its edges, though firm, yielded to pressure. I administered two scruples of powdered ergot. In twenty minutes it began to manifest its power, by producing pain in the lower part of the abdomen and back, which soon became severe, and continued without intermission for full half an hour, when she became quite easy. During this time there was but little bearing-down sensation, and I found, on examination, that neither the os uteri nor perineum had undergone any material change. She continued free from pain for nearly an hour, enjoying, in the interval, a little refreshing sleep. She was awaked by a return of the pains, which soon becoming severe and sensibly effective, the perineum was speedily on the stretch, and in less than half an hour my patient was delivered of a fine still-born child. After waiting full half an hour, the uterus in the interim shewing no disposition to throw off its contents, (although the belly and loins had been well rubbed, and the womb itself forcibly grasped from time to time with the hand, but without the effect of producing satisfactory contraction of the organ,) my patient began to flood considerably. Unable to trace the insertion of the fucus into the placental mass, I suspected there was hour-glass contraction; and, on introducing my hand, my suspicions were confirmed. I immediately withdrew it, gave her fifty drops of tincture of opium, and, after waiting half an hour, again passed my hand into the cavity of the uterus. The constriction was soon overcome; and as there was no adhesion, the after-birth was easily brought away. My patient ultimately did well, but no conception has since taken place." (P. 309.)

These cases are followed by some observations tending to shew that, since the employment of the ergot, the number of still-born children, both in this country and in America, has greatly increased. That retention of the placenta has also become much more frequent from the same cause may be gathered from the following extract.

"This event," (retention of the placenta,) says Mr. Chavasse, "has taken place, in my own practice, in twenty-three instances, during a period of *seven years.* In *eighteen* of the cases the children were still-born, and *in all* of these ergot had been administered. In the practice of my relative and partner, Mr. P. H. Chavasse, nine cases of still-born children have followed its use; and my experienced and valued friend, Mr. Jukes, a surgeon of the General Hospital in Birmingham, is so convinced of the fact, that he has long since abandoned its employment altogether." (P. 312.)

In a letter from Mr. Jukes which follows these remarks, it is stated that, in his early trials with the ergot, that gentleman "had no less than six cases of retention of the placenta almost in succession, three of which were well-marked cases of hour-glass contraction," five of the infants were still-born, and in four of the cases no pregnancy has since taken place. The os uteri was in each instance fairly dilated previous to the employment of the drug. We need not observe that the subject imperatively calls for the most careful and anxious investigation on the part of the profession, especially of such of its members as are employed in the management of this class of cases.

Dr. Paris Dick's paper on *the Unity of Organic Structure*, we pass over for the present, as it is not yet brought to a conclusion, and because the part which is here published contains little with which our readers are not already acquainted.

The highly interesting cases and observations of Dr. Barlow, which he entitles "*Records of Ovarian Tumours*," require a more extended notice. There are few of our medical brethren who have been much engaged in the practice of their profession but will agree with us in the opinion which we have been compelled to form, that the treatment of cases of ovarian disease has hitherto been not only unsatisfactory and delusive, but too frequently even destitute of any thing like either science in the selection of such remedial measures as have been employed, or steadiness or precision in the use of them. This has no doubt arisen from the intractable nature of the disease, from the length of time over which its duration extends, and from the conviction that those cases in which active measures were resorted to have seemed more speedily to arrive at a fatal termination than those in which nature has been almost entirely left to her own resources, or at least in which the physician has for the most part been content with palliating the symptoms as they become urgent. It was therefore with some curiosity that we turned to this article, being desirous of knowing the opinions of a physician so eminently qualified as Dr. Barlow, upon these obscure and unmanageable affections; and it was with no small gratification that we observed, that, by the application of the sound principles of pathology advocated in a work of the author's, not so generally known as it deserves to be,* to the treatment of this class of disorders, some at least of these cases, especially such as are of more recent origin, will be found to yield even under circumstances apparently of the most adverse description. We do not mean here to hold out the fallacious hope that any mode of treatment, however well conceived and adapted to the peculiar features of the case, and however judiciously carried into effect, can remedy that extreme state of disorganization of the ovary which dissection too often reveals; and Dr. Barlow is himself too close a reasoner to derive such an inference even from the extraordinary cases which he relates.

"Whoever has seen the mass of disease exhibited by an ovarium when it has become thoroughly disorganized, will readily admit the utter hopelessness of such total and extensive disorganization being remedied by any curative process with which we are familiar; any which our ordinary medicinal agents permit us the means of instituting or promoting. In cases which in the earlier stages yield to medical treatment, so far as to exhibit reduction or subsidence of tumour, we can have no demonstrative evidence of what was the kind or degree of organic change which produced the tumour; but it seems a fair inference, from the simple fact of such subsidence, that the tumour had not attained the utter perversion of all natural structure, by which the advanced disease is almost universally characterized. And this distinction, though resting on probability alone, is not unimportant; for it suggests, in the first place, that there is a stage of this disease produced by morbid actions alone, and in which these have not yet caused irremediable disorganization; and, secondly, it incites and encourages those remedial procedures by which the disorganized process may be arrested in that stage, in which alone there can be any well-founded hope of their proving effectual." (P. 392.)

In these sentiments we most fully concur, and if Dr. Barlow's "*Records*" were of no further value than to impress the truth, (for such we believe it to be,) that there is a stage in which this disease, however apparently unfavorable the external signs may be, has not proceeded to irremediable disorganization—a stage in which a hope may still be rationally entertained of restoring the normal condition of the organ, at least as far as the performance of its functions may be taken as evidence of such restoration,—they would still be of vast benefit, by the encouragement which they hold out to steady and persevering efforts directed towards subduing the formidable disease with which the practitioner has to contend.

Eight cases of ovarian disease are related. Of these, five terminated fatally, and, although of considerable interest and value, as affording good examples of the progress and pathology of the disease which it is the object of the paper to illustrate, need not now detain us. The remaining three, viz. cases 6, 7, and 8,† are instances of a favorable termination.

* An Essay on the Medicinal Efficacy and Employment of the Bath Waters, &c. by Edward Barlow, M.D., 8vo. Bath, 1822.

† Case 7 will be found at p. 422; but the number is omitted, probably by an error of the press.

We regret that we are unable to lay these cases before our readers in full, as they possess many points of great interest for consideration, and afford admirable illustrations of the judicious mode of treatment pursued by Dr. Barlow, and of patient perseverance in its application. In the first of these, (that of Sophia Wilkins,) from its first coming under treatment in September, 1828, until the complete removal of the symptoms of ovarian disease in June, 1832, the patient was bled from the arm eighteen times, losing 190 ounces of blood; 386 leeches were applied at thirty-one different periods; and she was twenty-one times cupped, 236 ounces of blood being taken. In addition to these measures, she had twelve blisters, and purgatives, and other parts of the antiphlogistic treatment were had recourse to. She has since her recovery been seen repeatedly by Dr. Barlow, under slight indisposition, but " there has been no renewal of ovarian derangement evinced by either tumour or pain."

We shall pass over the next case, that of Alice Kirkham, who has since married and become the mother of a healthy child, in order that we may insert an abstract of that of Mary Ann Scudamore, aged twenty-one, who, on the 25th of July, 1829, became an out-patient of the Bath United Hospital. She had at that time acute pains of the abdomen, with confined bowels, having previously suffered from diarrhœa. The kidneys subsequently becoming involved, and the urine suppressed, she was admitted an in-patient, and, after a course of depletory measures, with small doses of calomel and opium to affect the gums, she recovered, and was discharged well on the 1st September. The pains however returned, and she was readmitted on the 9th, there being then fulness in both iliac regions. On the 10th it is noted, " that these tumours were prominent, and separated by a depressed line extending from the umbilicus to the pubis." From this time to the 17th of November, when she was discharged better, she had occasional diarrhœa, with affections of the head and chest, for which, in addition to the depletory measures which were again had recourse to, appropriate remedies were employed. The gums were again affected with calomel and opium, and tartar emetic frictions used over the abdomen. On the 26th of December she again came under treatment, being then affected with cough and dyspnœa in addition to the abdominal symptoms. " The abdomen was more full, the breathing much oppressed, and there was œdema of the lower limbs." She was received into the house on the 6th of January, and suffered much from the abdominal symptoms, with incidental affections both of the head and heart, including several epileptic attacks, for which free depletion, general and local, blisters, calomel, and measures directed to relieve the incidental local affections, were assiduously employed. " On the 6th of April, the left hypogastric tumour had become so prominent, that a large caustic issue was established over it. From this time all her maladies subsided, and on the 4th of May she was discharged 'much relieved,' and made an out-patient." From this time until the 8th of September, when she was again admitted, she suffered much from diarrhœa, which however abated soon after her coming into the house. A cautious trial was then made of iodine, beginning with small doses, and this was persevered in for about a fortnight; when, in consequence of pains of the head and side, with inordinate action of the heart and hot skin, supervening, it was obliged to be laid aside. By bloodletting, &c. the disturbance was allayed, and she was discharged on the 9th of November, the report being — "general health much better, tumours remain." On the 22d of January, 1831, she was again admitted, requiring diligent and active use of remedies for successive affections of the head, chest, and abdomen. She was bled, cupped, and leeched repeatedly, had blisters several times, and iodine was again tried both externally and internally. "The hypogastric tumours advancing, caustic issues were formed over both, about the middle of April. From this time the contingent maladies, which had previously abated, continued to decline, and she quitted the hospital on the 20th of May, "much relieved." These issues remained open till the end of July, and were renewed towards the close of August, at her own desire; notwithstanding which, however, and the evident benefit derived from them, she suffered much from the various ailments she had previous undergone, with the addition of numbness and loss of power in the lower limbs, and also in one arm. "Under suitable treatment, however, this passed over, and improvement, general and progressive, ensued; the general health becoming reestablished, and *the tumours, at length, sensibly subsiding.* On the 16th of December she left the hospital, reported as "greatly relieved; tumours considerably reduced; slight diarrhœa alone remaining." From this time to the middle of May, 1833, she suffered much from occasional returns of her former symptoms, for which bleeding was still the remedy which could be most relied on; and, on the 1st of May, a caustic issue was once more formed in the left hypogastrium, in consequence of some evidences of renewed disturbance in the left ovarium. At the close of September she again appeared, affected with diarrhœa and pectoral complaints; but by this time "the ovarian tumours had entirely subsided, and such was her reliance on the amendment experienced in this respect, that, encouraged by the example of her fellow-sufferer, Alice Kirkham, she too had become a married woman." We need not present the history of the case further than to state in the words of Dr. Barlow that "on the 8th of November, (1834) she was safely delivered of three female children, who were baptized by the names of Faith, Hope, and Charity. She suckled the whole for the usual period, and both mother and offspring continued afterwards in perfect health, as I had repeated opportunities of witnessing." It may be right to add, that, "in the course of this protracted disease, she was bled forty-five times, to the extent of 384 ounces; was cupped twelve times, to 102 ounces; had 408 leeches applied; sixteen blisters; and six caustic issues, four of these being applied over the left ovarium, and two over the right."

We have dwelt so long upon the preceding memoirs, that we have little space to do more than refer to the cases which remain, although they are of much interest, particularly that of *successful cæsarean operation*, by Mr. Knowles, of Birmingham. For the same reason, were we not precluded by others of a different kind, we should be obliged to pass over Dr. Conolly's *biographical Notice of* Mr. Jennings; and we only allude to it here to express our regret at the loss which the profession has sustained by the decease of this ardent cultivator of medical science. The career of Mr. Jennings was indeed brought to an early close, so early as wellnigh to have prevented him from becoming known beyond the precincts of his own more immediate circle of attached friends. But to those of our professional brethren who reside in the midland counties, and more especially to many of his fellow members of the Provincial Association, we can appeal for the truth of the remark, that, bright as was the promise, the unwearied ardour with which he cultivated his profession as a science, no less than the talents natural and acquired which he brought to it, would, had his life and health been spared, more than have fulfilled anticipations so justly excited, but, alas, so fatally overthrown.

---

